# Viral Replication Rate Regulates Clinical Outcome and CD8 T Cell Responses during Highly Pathogenic H5N1 Influenza Virus Infection in Mice

**DOI:** 10.1371/journal.ppat.1001139

**Published:** 2010-10-07

**Authors:** Yasuko Hatta, Karen Hershberger, Kyoko Shinya, Sean C. Proll, Richard R. Dubielzig, Masato Hatta, Michael G. Katze, Yoshihiro Kawaoka, M. Suresh

**Affiliations:** 1 Influenza Research Institute, University of Wisconsin-Madison, Madison, Wisconsin, United States of America; 2 Department of Pathobiological Sciences, University of Wisconsin-Madison, Madison, Wisconsin, United States of America; 3 Division of Zoonosis, Department of Microbiology and Infectious Disease, Graduate School of Medicine, Kobe University, Kusunoki-cho, Chuo-ku, Kobe, Hyogo, Japan; 4 Department of Microbiology, School of Medicine, University of Washington, Seattle, Washington, United States of America; 5 Division of Virology, Department of Microbiology and Immunology and International Research Center for Infectious Diseases, Institute of Medical Science, University of Tokyo, Tokyo, Japan; Erasmus Medical Center, Netherlands

## Abstract

Since the first recorded infection of humans with H5N1 viruses of avian origin in 1997, sporadic human infections continue to occur with a staggering mortality rate of >60%. Although sustained human-to-human transmission has not occurred yet, there is a growing concern that these H5N1 viruses might acquire this trait and raise the specter of a pandemic. Despite progress in deciphering viral determinants of pathogenicity, we still lack crucial information on virus/immune system interactions pertaining to severe disease and high mortality associated with human H5N1 influenza virus infections. Using two human isolates of H5N1 viruses that differ in their pathogenicity in mice, we have defined mechanistic links among the rate of viral replication, mortality, CD8 T cell responses, and immunopathology. The extreme pathogenicity of H5N1 viruses was directly linked to the ability of the virus to replicate rapidly, and swiftly attain high steady-state titers in the lungs within 48 hours after infection. The remarkably high replication rate of the highly pathogenic H5N1 virus did not prevent the induction of IFN-β or activation of CD8 T cells, but the CD8 T cell response was ineffective in controlling viral replication in the lungs and CD8 T cell deficiency did not affect viral titers or mortality. Additionally, BIM deficiency ameliorated lung pathology and inhibited T cell apoptosis without affecting survival of mice. Therefore, rapidly replicating, highly lethal H5N1 viruses could simply outpace and overwhelm the adaptive immune responses, and kill the host by direct cytopathic effects. However, therapeutic suppression of early viral replication and the associated enhancement of CD8 T cell responses improved the survival of mice following a lethal H5N1 infection. These findings suggest that suppression of early H5N1 virus replication is key to the programming of an effective host response, which has implications in treatment of this infection in humans.

## Author Summary

Outbreaks of avian influenza (AI) viruses have continued in chickens in Southeast Asia, coupled with regular instances of direct bird to human transmission, with extremely high case fatality rates. The mechanisms underlying the disease pathogenesis and high mortality rate in humans are not well understood. In particular, we lack information on the development and/or failure of adaptive immune responses during AI infection. Our studies in mice have linked the pathogenicity of AI viruses to the virus' rate of replication in the lungs. Surprisingly, a strong T cell response was triggered by the infection, but virus-specific T cells were ineffective in controlling the rapidly replicating virus. The extremely high rate of AI virus replication likely outpaces and overwhelms the developing immune response. However, administration of anti-viral drugs, only early in the infection slowed viral replication, enhanced the number of effector CD8 T cells in the lung, and promoted survival and recovery from infection. These findings highlight the role of viral replication rate in pathogenesis and underscore the importance of controlling viral replication as an adjunct to immunotherapies in the treatment of this infection in humans.

## Introduction

Severe outbreaks of highly pathogenic avian influenza (AI) H5N1 viruses in poultry continue to occur and are often coupled with reports of direct bird-to-human viral transmission. Between 2003 and 2009, 406 confirmed human cases of AI H5N1 were reported, with a fatality rate of >60% (http://www.who.int/csr/disease/avian_influenza/country/cases_table_2010_01_28/en/index.html). Although sustained human-to-human transmission has not yet occurred, there is increasing concern that these H5N1 AI viruses might acquire the ability to transmit efficiently between humans and cause a pandemic. The high virulence of H5N1 viruses in humans can be attributed to either a delay in development or the ineffectiveness of innate and/or adaptive immune mechanisms to control the infection in a timely fashion. However, little information exists on the dynamics of adaptive immune responses to H5N1 viruses during a primary infection, which constitutes a staggering gap in our understanding of the pathogenesis of lethal H5N1 infection in humans.

The adaptive immune response to seasonal influenza viruses has been extensively characterized using a murine model of intranasal (I/N) infection with mouse-adapted influenza viruses [1], [2], [3], [4], [5], [6]. Elicitation of a potent CD8 T cell response is of critical importance in resolving a primary influenza virus infection in mice [1], [3], [4], [7]. However, both CD8 T cells and antibodies might be required to clear highly pathogenic influenza viruses [8]. Mouse-adapted influenza viruses elicit robust CD8 T cell responses in the respiratory tract, which typically peak at day 10 after infection [5], [6]. Effector CD8 T cells control influenza virus replication by cytolytic mechanisms that require Fas and/or perforin [2]. In addition to their role in viral clearance, CD8 T cells are also implicated in mediating immune-mediated lung injury following influenza virus infection [9], [10], [11]. Pertaining to primary infection with H5N1 viruses, we do not yet know whether CD8 T cell responses are induced in the respiratory tract, or whether virus-specific CD8 T cells play a protective or immunopathologic role during a primary H5N1 infection. A high viral load is one of the hallmarks of a fatal H5N1 infection in humans [12], but the effect of high-level H5N1 virus replication on the emergence of CD8 T cell responses in the respiratory tract has not been studied.

In this study, using two human isolates of H5N1 viruses that differ in their pathogenicity in mice, we have systematically examined the following: 1) the relationship between the speed of H5N1 virus replication and viral pathogenicity on the dynamics of CD8 T cell responses, 2) whether ineffective control of H5N1 virus infection is related to the suppression of virus-specific CD8 T cell responses, 3) the effect of CD8 T cell deficiency on host survival, and 4) the effect of anti-viral therapy on CD8 T cell responses. Findings from these studies have provided novel insights into the virus/immune system interactions during an H5N1 infection from the standpoint of viral pathogenesis, immune control of viral replication, and immunopathology.

## Results

### The pathogenicity of H5N1 viruses in mice is associated with accelerated viral replication in the lungs

**Figure 1:**
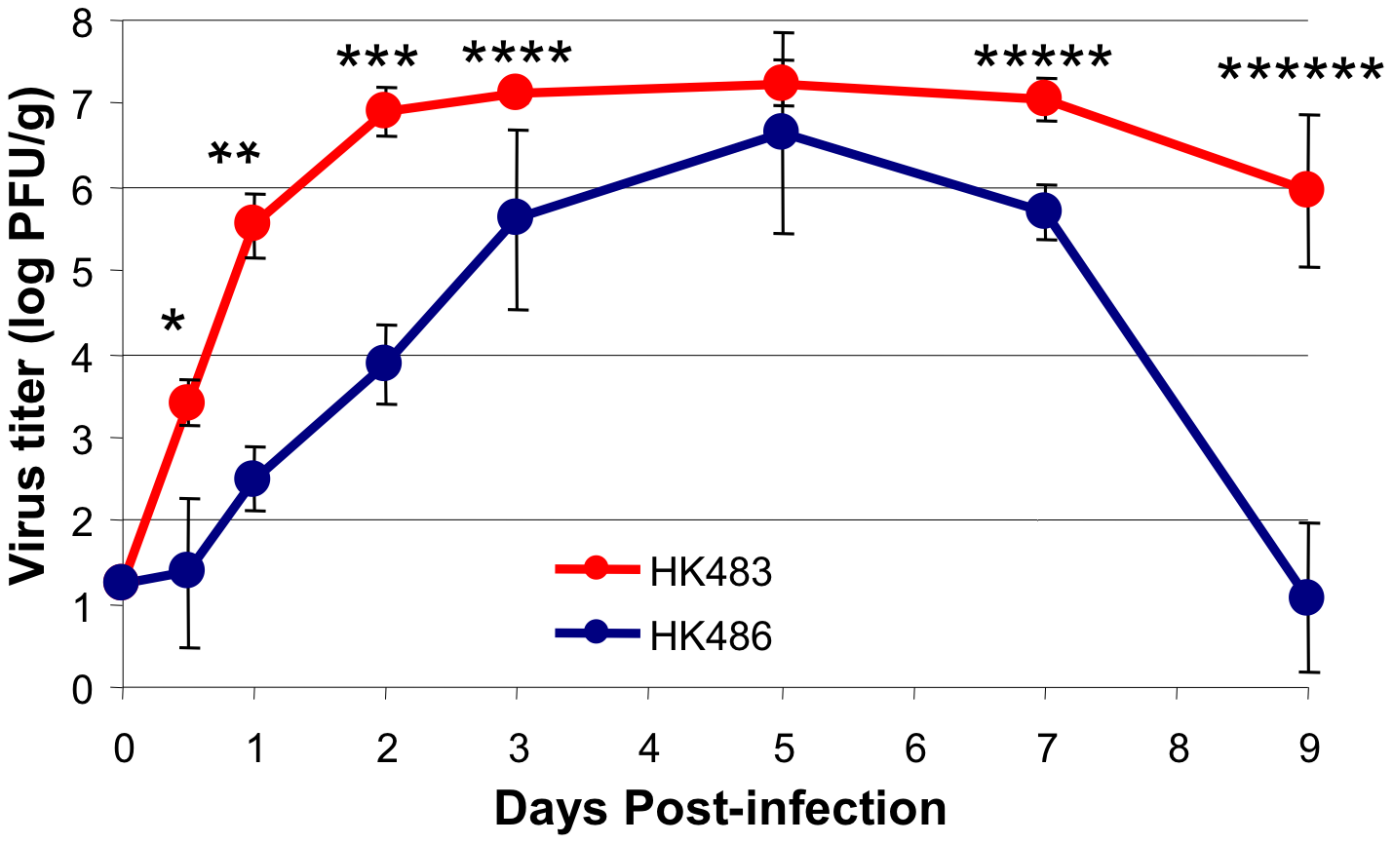
Virus replication kinetics in lungs of mice infected with H5N1 viruses HK483 or HK486. At indicated days after I/N infection with 18 PFU of HK483 or HK486 virus, mice were euthanized and lung virus titers were determined by plaque assay on MDCK cells. The data are the average titers from 3 mice ± SD at each time point. Data is representative of 2 independent experiments. * P = 0.01; ** P = 0.0005; *** P = 0.0005; **** P = 0.02; ***** P = 0.004; ****** P = 0.002.

Unlike seasonal strains of influenza viruses, human isolates of H5N1 viruses readily replicate in other mammals, including mice, without prior adaptation and induce varying levels of pathogenicity [13]. Experimental infections of mice with the H5N1 viruses have led to the identification of viral determinants of pathogenicity [13], [14], [15], [16], [17]. Although high cleavability of hemagglutinin is essential to cause a lethal infection, a single amino acid residue in the PB2 protein controls the pathogenic potential of these AI viruses in mice [13]. An extremely low-dose infection of mice with the A/Hong Kong/483/97 (HK483) virus that has a Lys at position 627 of the PB2 protein induces a lethal infection (a dose of virus that kills 50% of infected mice (MLD_50_) of 1.8 plaque-forming units [PFU]), whereas the A/Hong Kong/486/97 (HK486) virus that has a Glu at position 627 of PB2 is less pathogenic (MLD_50_ of 7.6×10^3^ PFU). To determine whether the two viruses differ in their rate of viral replication in vivo in the respiratory tract, we performed a detailed kinetic analysis of viral titers in the lungs of HK483- and HK486-infected mice (**Figure 1**). Although mice were infected with the same dose of both viruses and reached comparable maximum titers, viral growth kinetics in the lungs were dramatically different. The HK483 virus replicated at a remarkable pace within the first 24 hours, and the coefficient of expansion was calculated to be ∼4.3 log PFU/day; peak virus titers of ∼10^7^ PFU/gram were attained in the lungs within 48 hours after infection. In striking contrast, the coefficient of expansion for the less pathogenic HK486 virus in the first 24 hours was only ∼1.3 log PFU/day, and peak titers in the lungs were not attained until 5 days after infection. Thus, the speed of early viral replication in the lungs might be a necessary and distinguishing trait of highly pathogenic AI viruses to rapidly reach high titers and potentially overwhelm the host immune responses.

The enhanced replication of the HK483 virus could be related to the virus's ability to evade the innate immune mechanism(s), especially the type I IFN pathway [18]. However, microarray analysis showed that the induction of IFN-β and interferon-stimulated genes is greater in the lungs of HK483-infected mice compared to HK486-infected mice at 48 hours after infection (**Figure S1**). To examine whether Type I IFNs play any role in controlling infection with HK483, we infected groups of wild type C57BL/6 (n = 5) and Type I IFN receptor-deficient (IFNRI−/−) mice (n = 5) with 18 PFU of HK483 virus. Upon infection with the HK483 virus, all wild-type mice survived at least until day 7 after infection, but 4 of 5 IFNRI−/− mice died by day 5 postinfection (PI), and the remaining IFNRI−/− mouse died on day 7 PI. These data suggested that the type I IFN pathway is induced and functional in HK483-infected mice, which is consistent with a recently published report [19].

### CD8 T cell responses to H5N1 influenza viruses in mice

**Figure 2:**
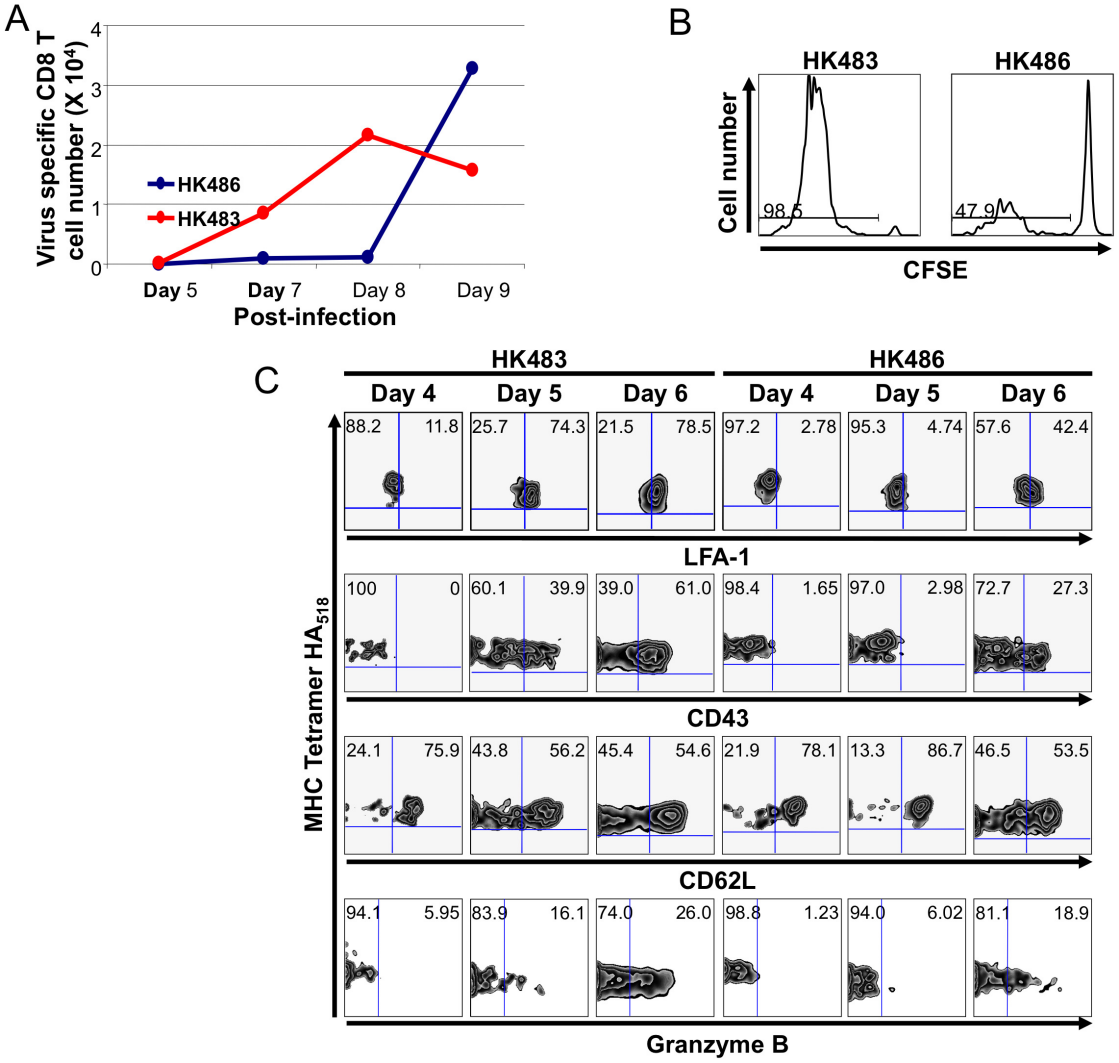
CD8 T cell responses in the lung airways of mice infected with H5N1 viruses. Panel A, Groups of BALB/C mice were infected I/N with 18 PFU of HK483 or HK486 virus. At the indicated days after infection, pooled cells from broncoalveolar lavage of 3 mice were collected. Cells were stained with anti-CD8, anti-LFA-1, and K^d^/NP147 MHC I pentamers, and pentamer-binding LFA-1^Hi^ CD8 T cells were quantified by flow cytometry. The data are representative of two independent experiments. Panel B, CFSE-labeled Thy1.1^+ve^ TCR transgenic CL-4 CD8 T cells were adoptively transferred in to congenic Thy1.2/BALB/c mice, and infected I/N with 18 PFU of HK483 or HK486 virus 24 hours later. At day 5 after infection, cells from deep cervical lymph nodes were stained with anti-CD8, anti-Thy1.1, and K^d^/HA518 tetramers. The histograms are gated on CD8^+^/Thy1.1^+^ MHC-I tetramer-binding cells, and the numbers are the percentage of divided cells of total gated cells. Data are representative of analysis of pooled cells from 3 mice/group. Panel C, As in panel B, CL-4 CD8 T cells were adoptively transferred into BALB/c mice, and infected with HK483 or HK486 virus. At the indicated time points after infection, cells from the deep cervical lymph nodes were stained with anti-CD8, anti-Thy1.1, K^d^/HA518 tetramers, anti-LFA-1, anti-CD43, anti-CD62L, or anti-granzyme B. The flow cytometry plots are gated on CD8^+^/Thy1.1^+^ MHC-I tetramer-binding cells, and the numbers are the percentage of LFA-1^LO/HI^, CD43^LO/HI^, or CD62^LO/HI^, or granzyme B^LO/HI^ of total gated cells.

To understand the relationship of viral pathogenicity and/or rapid viral replication rate to the development of adaptive immunity, we infected BALB/c mice I/N with the HK483 or HK486 virus and studied the evolution of virus-specific CD8 T cell responses in the respiratory tract. Sequence comparisons showed that the K^d^-restricted CD8 T cell epitope NP147 of the PR8 virus was conserved in both HK483 and HK486 viruses. In mice infected with the less pathogenic HK486 virus, high numbers of CD8 T cells in the lung airways were not detectable until day 8 PI but rapidly accumulated within the next 24 hours (**Figure 2A**). The kinetics of the CD8 T cell response to HK486 were similar to those of mouse-adapted human influenza viruses [5], [6]. Surprisingly, in HK483-infected mice, virus-specific CD8 T cells were detectable earlier, at day 7 PI, and peak numbers were attained at day 8 PI. It is noteworthy that the peak numbers of NP147-specific CD8 T cells in HK483-infected mice attained at day 8 PI were lower, as compared to those in HK486-infected mice (day 9 PI). Additionally, CD8 T cells in HK483-infected mice appear to have initiated contraction between days 8 and 9 PI, when the number of CD8 T cells continued to increase in the respiratory airways of HK486-infected mice (**Figure 2A**). Similar contraction in the number of CD8 T cells was seen in the lungs of HK483-infected mice between days 8 and 9 PI in a separate experiment (data not shown).

In order to track the early events of CD8 T cell activation in the draining lymph nodes (DLNs), we adoptively transferred carboxyfluorescein succinimidyl ester (CFSE)-labeled influenza HA518-specific Clone 4 (CL-4) TCR transgenic CD8 T cells into congenic BALB/c mice [5], [6], which were subsequently infected I/N with 18 PFU of HK483 or HK486 virus. By day 5 PI, >90% of CL-4 CD8 T cells had divided several times in the DLNs of HK483-infected mice (**Figure 2B**), and a substantial fraction of these cells also exhibited markers of activation (LFA-1^HI^, CD43^Hi^, and CD62L^Lo^) (**Figure 2C**). In contrast, in the DLNs of HK486-infected mice, only <50% of CL-4 CD8 T cells had proliferated by day 5 PI, and these cells did not upregulate expression of LFA-1 or CD43 until day 6 PI. The increased percentage of proliferated CL-4 CD8 T cells in the lymph nodes of HK483-infected mice was not linked to reduced trafficking of these cells out of the lymph nodes into the lungs because, the number of CL-4 CD8 T cells in the BAL of HK483-infected mice (2.9–4.1×10^3^) were higher than in the BAL of HK486-infected mice (1.0–1.4×10^3^) at day 5 PI. In addition to increased proliferation, a larger percentage of CL-4 CD8 T cells expressed granzyme B in HK483-infected mice at day 6 PI compared to those in HK486-infected mice (**Figure 2C**). Thus, CD8 T cells underwent accelerated activation in the DLNs of mice infected with the HK483 virus compared to those in HK486-infected mice. The early activation of virus-specific CD8 T cells in lymph nodes of HK483-infected mice corresponds with the faster replication kinetics of the virus in the respiratory tract.

### Viral pathogenicity regulates CD8 T cell responses to H5N1 influenza viruses

**Figure 3:**
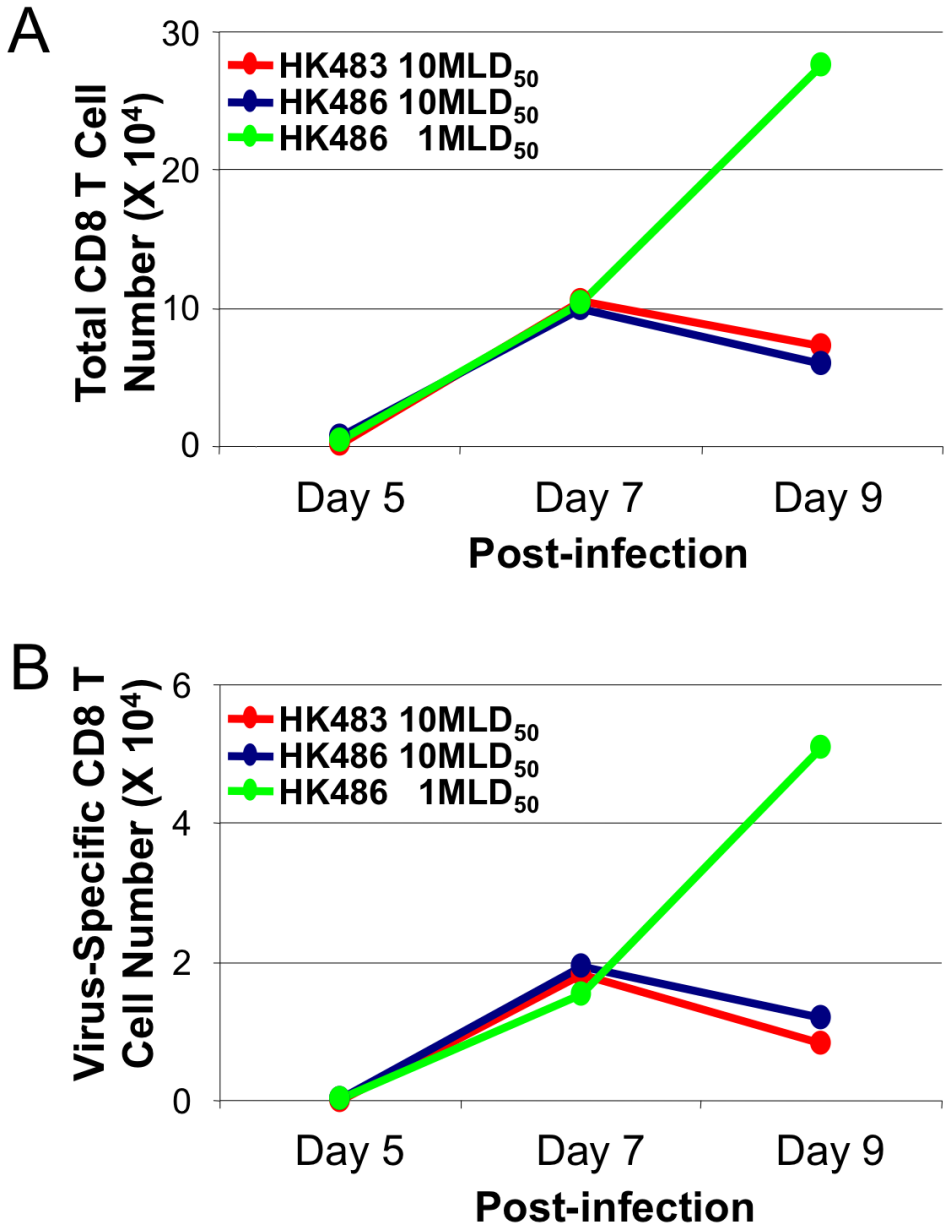
Premature contraction of CD8 T cells in mice infected with lethal dose of highly pathogenic H5N1 influenza viruses. BALB/c mice were I/N inoculated with 1 MLD_50_ or 10 MLD_50_ of HK486 or 10 MLD_50_ of HK483 virus. At indicated days after infection, pooled cells from the BAL of 3 mice were stained with anti-CD8, anti-LFA-1, and K^d^/NP147 pentamers. The numbers of activated LFA-1^Hi^ CD8 T cells (Panel A) or NP147-specific LFA-1^Hi^ CD8 T cells (Panel B) were quantified by flow cytometry. Data are from one of two independent experiments.

The pathogenicity of influenza viruses in the experimental mouse model has been defined based on MLD_50_. Next, we determined whether the accelerated kinetics of CD8 T cell contraction is related to the clinical outcome of infection, i.e., lethality. Typically, varying the infecting dose alters the disease process and clinical outcome of influenza viruses, but this procedure is not feasible with the HK483 virus because of the extremely low MLD_50_ of 1.8 PFU. Therefore, we examined the effect of viral dose (based on MLD_50_) on the kinetics of CD8 T cell contraction by infecting BALB/c mice with 1 (4.6×10^3^ PFU) or 10 MLD_50_ (4.6×10^4^ PFU) of the HK486 virus. As controls, mice were infected with 10 MLD_50_ of the HK483 virus (18 PFU). As shown in **Figure 3**, premature contraction of total and NP147-specific CD8 T cells occurred in mice infected with 10 MLD_50_ of the HK483 or HK486 virus, but not in mice infected with 1 MLD_50_ of the HK486 virus. Thus, regardless of the H5N1 virus strain used, the pathogenicity of H5N1 viruses in mice (which is a function of infecting dose for the HK486 virus) regulated the dynamics of CD8 T cell contraction in the respiratory tract following infection with H5N1 viruses.

### Highly pathogenic H5N1 influenza viruses trigger accelerated apoptosis of CD8 T cells and lung damage by BIM-dependent mechanisms

**Figure 4:**
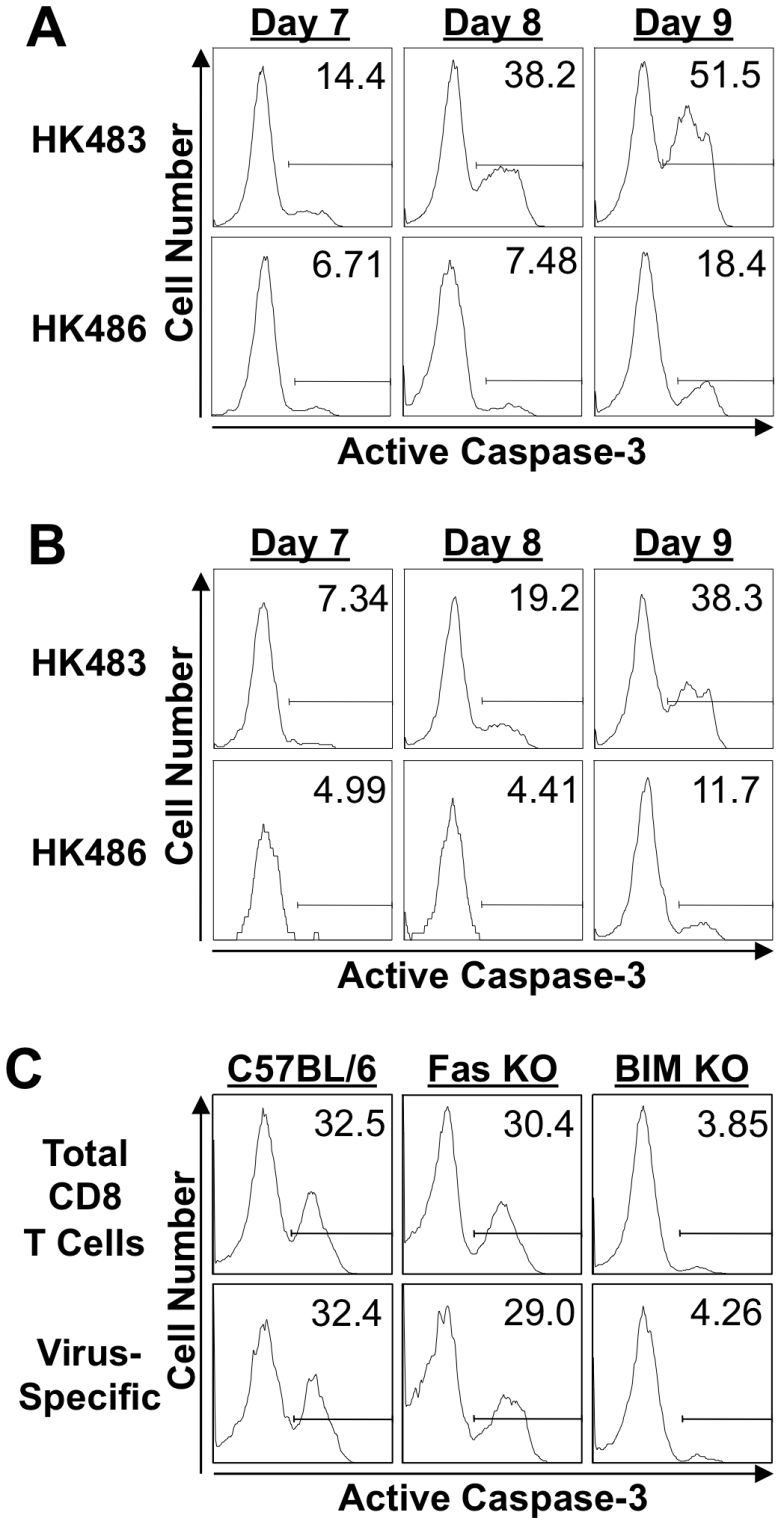
Apoptosis of CD8 T cells in mice infected with H5N1 viruses. A and B, BALB/c mice were infected with 18 PFU of HK483 or HK486. At the indicated days after infection, the percentages of total CD8 T cells (Panel A) and K^d^/NP147-specific CD8 T cells (Panel B) expressing active form of caspase-3 were determined by flow cytometry; cells in the BAL were stained with anti-CD8, K^d^/NP147 pentamers, and anti-active caspase-3. Panel C, C57BL/6 (+/+), Fas-deficient (Fas KO) or BIM-deficient (BIM KO) mice (n = 3) were infected with 18 PFU of HK483. On the eighth day after infection, the percentages of total CD8 T cells and of D^b^/PA224-specific CD8 T cells expressing active form of caspase-3 in the BAL were determined as in panels A and B above.

Next, we determined whether infection with the highly pathogenic H5N1 virus caused premature contraction of CD8 T cells by affecting cellular apoptosis. Following infection of BALB/c mice with 18 PFU of HK483 or HK486 viruses, apoptosis of CD8 T cells in the lung was assessed at days 7, 8, and 9 after infection by staining for active caspase 3. At all time points, the fraction of apoptotic CD8 T cells in the lungs of HK483-infected mice was two- to four-fold higher than in HK486-infected mice (**Figures 4A and 4B**). These findings suggested that infection with highly pathogenic AI viruses induces accelerated apoptosis and premature contraction of CD8 T cells in the respiratory tract.

Two distinct pathways of caspase-dependent cellular apoptosis have been described: the intrinsic and extrinsic pathways [20], [21]. The intrinsic apoptotic pathway is initiated following activation of the pro-apoptotic BH3-only proteins, such as Bcl-2-interacting mediator of death (BIM). On the other hand, interaction between death receptors and their ligands, such as Fas and Fas ligand, triggers the extrinsic pathway of cellular apoptosis. To determine which pathway of cellular apoptosis is triggered in CD8 T cells by the highly pathogenic AI virus, we infected wild-type C57BL/6 (+/+), Fas-mutant lpr/lpr (Fas KO), and BIM-deficient (BIM KO) mice with the HK483 virus. At day 8 PI, we quantified the number of apoptotic active caspase 3^+ve^ CD8 T cells in the BAL of HK483-infected mice (**Figure 4C**). As expected, a substantial fraction of CD8 T cells was apoptotic in the lungs of C57BL/6 mice, and Fas deficiency did not significantly affect apoptosis of CD8 T cells induced by highly pathogenic HK483 infection. Notably, the percentage of apoptotic CD8 T cells was reduced by ∼90% in BIM KO mice compared to C57BL/6 or Fas KO mice. These data suggested that the apoptosis of CD8 T cells induced by highly pathogenic AI viruses is triggered by the intrinsic pathway of cellular apoptosis. As a consequence of reduced apoptosis in the absence of BIM activity, the numbers of PA224-specific CD8 T cells in the BAL of BIM KO mice (5.4×10^4^) were higher than in +/+ mice (3.6×10^4^).

**Figure 5:**
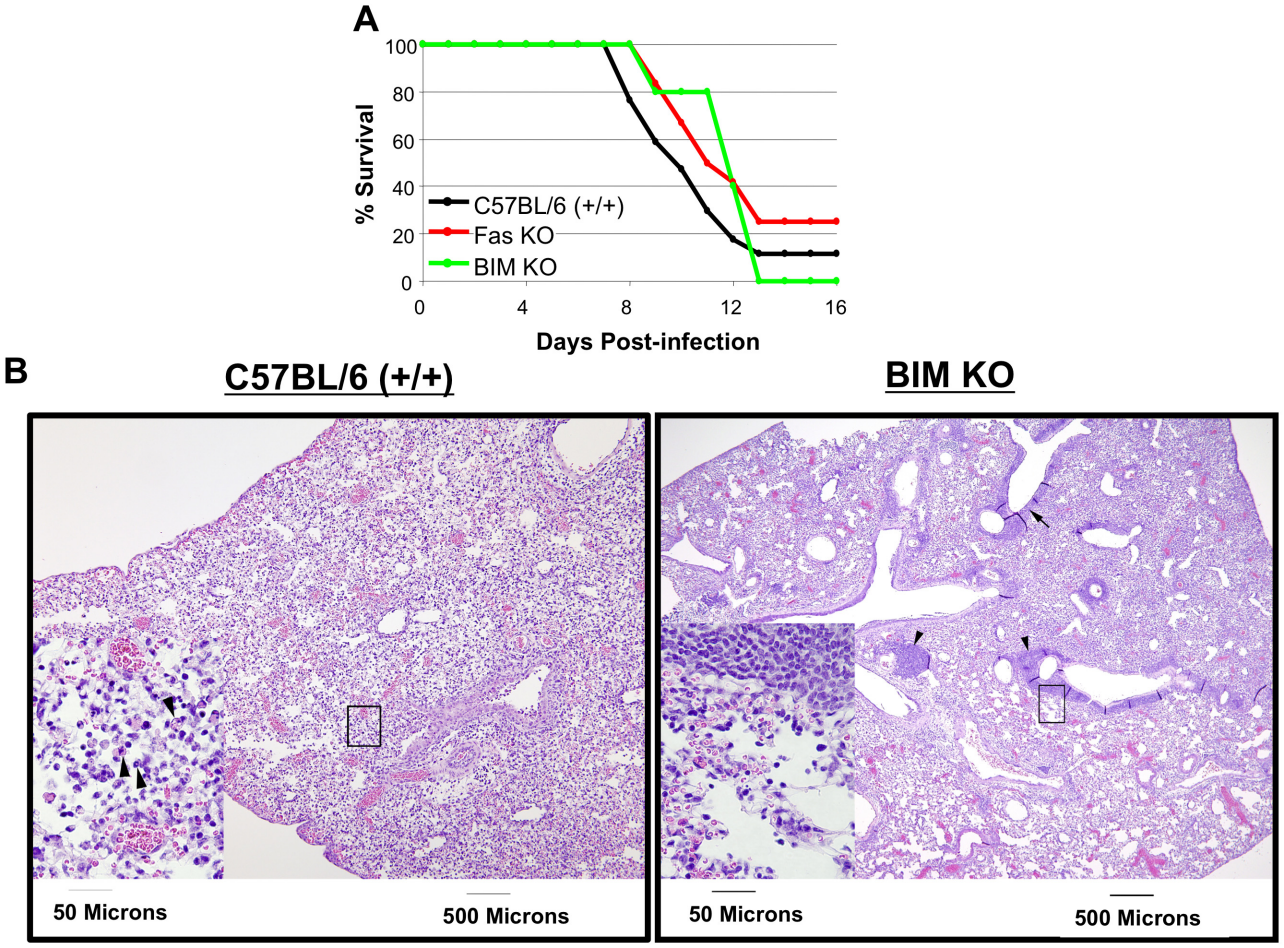
Survival of C57BL/6, Fas KO, and BIM KO mice after inoculation with highly pathogenic HK483 virus. C57BL/6 (+/+; n = 17), Fas KO (n = 12), or BIM KO (n = 5) mice were I/N inoculated with 18 PFU of HK483 virus and mouse survival was monitored for 16 days (Panel A). Panel B, At day 8 after infection, lungs from infected mice were examined for histopathological changes. Arrows indicate apoptotic cells in lung section from +/+ mouse or lymphocytic infiltrates adjacent to the bronchioles in lung section from BIM−/− mice.

Because BIM deficiency protected against CD8 T cell apoptosis, we next examined whether the loss of BIM would also improve survival of HK483-infected mice. Groups of +/+, Fas KO, and BIM KO mice were infected with the HK483 virus as above, and their survival was monitored daily. As shown in **Figure 5A**, a majority of +/+ mice infected with HK483 succumbed to infection by day 12 PI. Likewise, BIM KO and Fas KO mice also succumbed to HK483 infection, albeit with a slight delay. Neither BIM nor Fas deficiency significantly affected viral titers in the lungs (**Figure S2**). These data suggested that BIM deficiency-induced enhancement of virus-specific CD8 T cell responses is insufficient to enhance survival following a highly pathogenic H5N1 virus infection.

Next, we assessed whether BIM deficiency affected HK483 virus-induced cell damage in the lungs (**Figure 5B**). At day 8 after infection with the HK483 virus, the lung pathology in +/+ mice was characterized by extensive cellular necrosis and tissue disruption of medium-sized blood vessels and bronchioles, which was associated with infiltration of inflammatory cells composed mostly of neutrophils. Additionally, apoptotic cells were frequently observed in the lungs of +/+ mice. In striking contrast, in the lungs of BIM−/− mice, cellular necrosis was less pronounced and the tissue integrity was more intact, with less frequent apoptotic cells. Notably, the lungs of BIM−/− mice contained lymphocytic infiltrates in the connective tissues near medium-sized blood vessels and, more prominently, adjacent to the bronchioles (**Figure 5B**). Based on these findings, we infer that HK483-induced lung pathology is at least in part mediated by BIM-dependent mechanisms.

### A neuraminidase inhibitor prevents accelerated CD8 T cell contraction and enhances survival during a highly pathogenic H5N1 influenza virus infection

**Figure 6:**
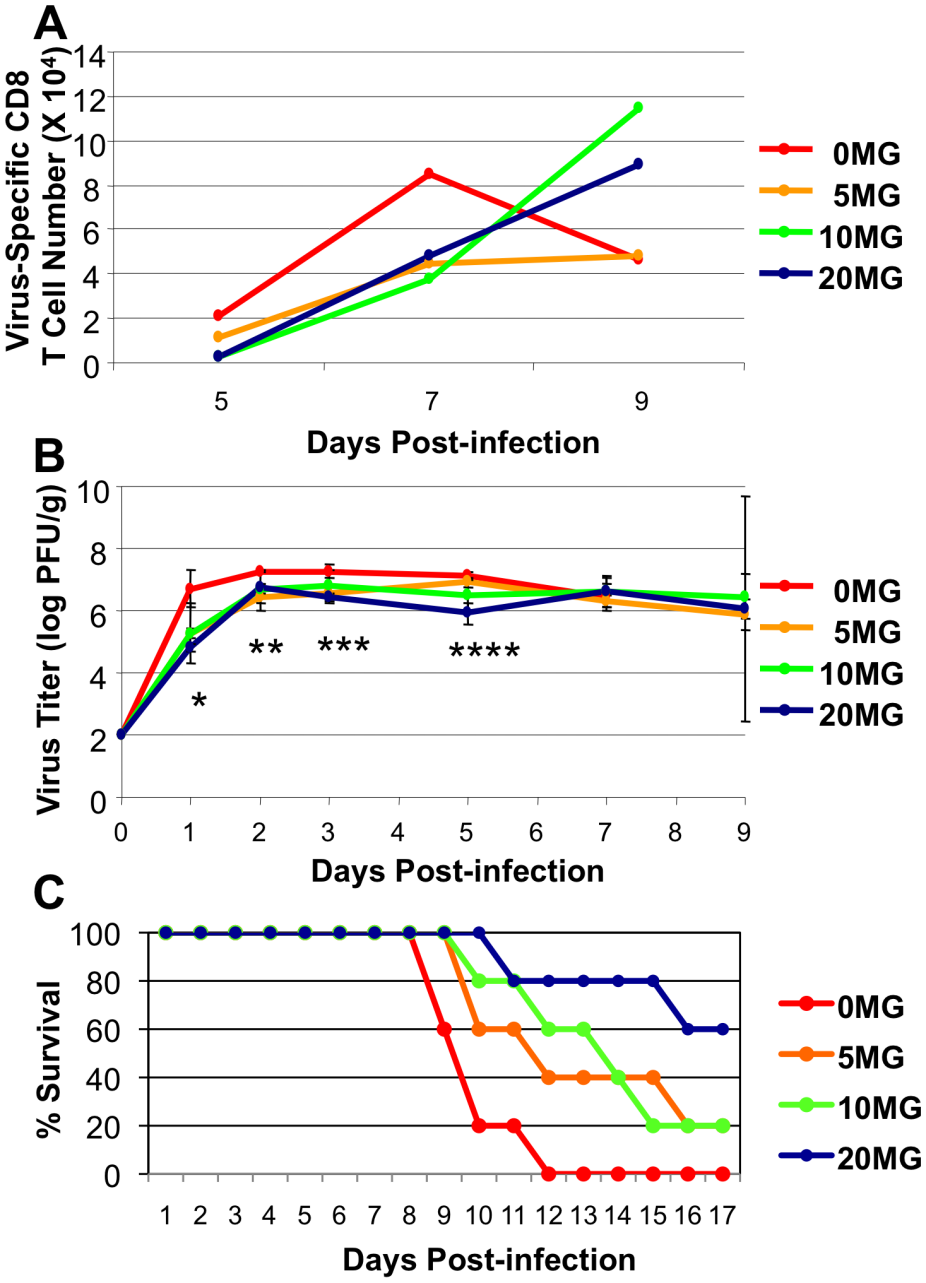
Oseltamivir therapy protects against accelerated activation and contraction of CD8 T cells following infection of mice with HK483 virus. C57BL/6 mice were treated daily with indicated doses of oseltamivir (mg/Kg body weight) from −1 day to 3 day relative to infection with 100 PFU of HK483. Panel A, At days 5, 7, and 9 post-infection, BAL samples from 3 mice/dose were pooled and the number of CD8 T cells specific to CTL epitope PA224 were determined by flow cytometry. Panel B, At days 1, 2, 3, 5, 7, and 9 PI, 3 mice were euthanized at each time point/dose and lung virus titers were determined; error bars indicate SD. * P = 0.03 between 0 mg and 5 mg doses and P = 0.06 between 0 mg and 10 mg doses; ** P = 0.0003 between 0 mg and 5 mg doses, P = 0.08 between 0 mg and 10 mg doses, and P = 0.005 between 0 mg and 20 mg doses; *** P = 0.02 between 0 mg and 5 mg doses, P = 0.006 between 0 mg and 20 mg doses; **** P = 0.12 between 0 mg and 5 mg doses and P = 0.07 between 0 mg and 10 mg doses, and P = 0.004 between 0 mg and 20 mg doses. Panel C, Effect of oseltamivir treatment on survival of HK483-infected mice (n = 5/group). Data in panels B and C is representative of two independent experiments.

The neuraminidase inhibitor, oseltamivir phosphate, is an effective treatment for influenza A virus infection in humans if given early in the infection [22], [23], [24]. Treatment with oseltamivir reduces viral load and protects mice against a lethal H5N1 virus infection [25], [26]. It was of interest to determine whether a high rate of virus replication in HK483-infected mice, especially early in the infection could 1) lead to early activation and contraction of virus-specific CD8 T cells in the lung airways and 2) outpace and overwhelm the CD8 T cell response. Additionally, the effects of oseltamivir treatment on the adaptive immune response to H5N1 infection have not been examined. Therefore, we asked whether the reduction of virus replication by oseltamivir protected against the accelerated activation and contraction of CD8 T cells following infection of mice with the highly pathogenic HK483 virus. Mice that were infected with the HK483 virus were treated with graded doses of oseltamivir only early in the infection, and virus-specific CD8 T cells were quantified at days 7, 8, and 9 after infection. As expected, CD8 T cells in control vehicle-treated mice underwent contraction between days 8 and 9 PI (**Figure 6A**), but oseltamivir treatment at doses of 10 or 20 mg, but not 5 mg, mitigated contraction and led to a substantive increase in the number of virus-specific CD8 T cells in the lung airways of HK483-infected mice between days 8 and 9 PI. Notably, **Figure 6B** shows that oseltamivir treatment reduced viral titers, especially early in the course of the infection, regardless of the dose administered, but mouse survival was extended or increased only at doses of 10 and 20 mg, which suggested that suppression of early viral replication alone might be necessary but not be sufficient to enhance mouse survival. However, reduced viral titers coupled with enhanced CD8 T cell responses were associated with extended or improved survival.

### Effect of CD8 T cell deficiency on the survival of mice following a highly pathogenic H5N1 virus infection

**Figure 7:**
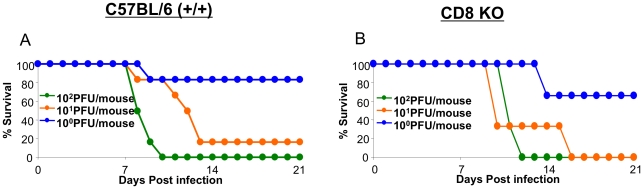
Effect of CD8 T cell deficiency on survival of mice infected with H5N1 viruses. C57BL/6 (+/+) or CD8-deficient (CD8 KO) mice were infected intranasally with the indicated doses of HK483 virus (Panels A and B). Three to six mice were infected at each dose and mouse survival was observed for 21 days.

Our studies showed that infection with the highly pathogenic HK483 virus elicited a readily detectable CD8 T cell response but failed to effectively control viral replication. Because there is evidence supporting a role for CD8 T cells in augmenting lung pathology following infection with seasonal influenza viruses [9], [10], [11], we questioned whether CD8 T cells contribute to the lethality induced by infection with highly pathogenic H5N1 viruses. Groups of +/+ and CD8-deficient (CD8 KO) mice were infected with graded doses of the HK483 virus, and mouse survival was monitored (**Figure 7**). As shown in **Figures 7A and 7B**, there was no difference in survival between HK483-infected +/+ and CD8 KO mice. These data suggested that the loss of a CD8 T cell response does not provide either a survival advantage or a disadvantage to mice infected with the highly pathogenic HK483 virus.

## Discussion

Until the AI epidemic of 1997, it was assumed that purely AI viruses could not cause a lethal disease in humans. However, since 1997, recurring instances of direct transmission of AI viruses from birds to humans have dismissed this assumption [14], [27], [28]. A unique feature of these H5N1 viruses is their ability to replicate to high levels in the lungs of several mammalian species, including humans, without adaptation [15], [29], [30]. Although a high viral load and hypercytokinemia are recognized hallmarks of fatal AI infections in humans [12], we still lack crucial information on the kinetics, magnitude, and nature of the adaptive immune response to these infections. In this study, we have examined the relationship of viral replication kinetics in the lungs and viral pathogenicity to the dynamics of virus-specific CD8 T cell responses to AI viruses in mice. We found that the extreme pathogenicity of H5N1 viruses is directly linked to the high viral replication rate and the consequent production of peak steady-state viral titers in the lungs within 48 hours after infection. Interestingly, we found that lethal H5N1 infection in mice stimulates a robust, virus-specific CD8 T cell response in the respiratory tract, but these CD8 T cells fail to control viral replication and undergo early contraction. The prevention of CD8 T cell contraction did not alter the survival of infected mice, but inhibition of neuraminidase activity and viral replication by therapeutic intervention mitigated the premature contraction of CD8 T cells and enhanced mouse survival following a lethal H5N1 infection. These findings suggest that the ability of H5N1 viruses to overwhelm and/or undercut the sustenance of the anti-viral CD8 T cell response and cause a lethal pulmonary infection is linked to a high viral replication rate, especially early in the infection. These findings further our understanding of the pathogenesis of H5N1 viruses, which should have implications on the development of novel therapies and prophylaxis for H5N1 infection in humans.

The infection of mice with mouse-adapted strains of influenza viruses elicits strong CD8 T cell responses in the respiratory tract, and there is ample evidence indicating an important role for CD8 T cells in the viral control of a primary influenza virus infection [3], [4], [7]. In contrast to a sublethal infection, inoculation of mice with high doses of mouse-adapted influenza virus leads to apoptosis of virus-specific CD8 T cells and lethal pulmonary injury [31]. Moreover, based on an analysis of gene expression in the lungs of mice infected with highly pathogenic H5N1 viruses, T cell activation might be impaired during an H5N1 virus infection [32]. However, we showed that the infection of mice with a highly pathogenic H5N1 virus elicits a readily detectable CD8 T cell response, which suggests that the initial events of T cell priming, including trafficking of dendritic cells to the DLN and antigen processing/presentation, are intact in H5N1 virus-infected mice. While virus-specific CD8 T cells continued to accumulate in the lung airways of mice until at least day 9 after infection with the less pathogenic HK486 virus, CD8 T cells in HK483-infected mice exhibited a decline after day 8 PI due to BIM-dependent apoptosis. The BIM-dependent intrinsic pathway of apoptosis of activated CD8 T cells appears to be unique to highly pathogenic H5N1 AI viruses because a high-dose infection of mice with mouse-adapted epidemic strains of the influenza virus induced CD8 T cell apoptosis that was dependent upon Fas/FasL interactions [31]. Highly pathogenic AI viruses are known to trigger hyperinduction of TNF-related apoptosis inducing ligand (TRAIL) in macrophages and cause T cell apoptosis in vitro [33]. Because TRAIL-induced apoptosis is BIM-dependent [34], [35], [36], we propose that apoptosis of activated CD8 T cells in HK483-infected mice might be triggered by interactions between macrophage-derived TRAIL and its receptors on CD8 T cells. It should be noted that our experiments did not test whether BIM triggered apoptosis of CD8 T cells by T cell intrinsic mechanisms. It is possible that reduced CD8 T cell apoptosis in BIM-deficient mice was an indirect effect, possibly linked to increased survival of dendritic cells [37]. Do differential direct infection of CD8 T cells by HK483 and HK486 viruses explain differences in CD8 T cell apoptosis? Studies of apoptosis in the lungs of mice infected with HK483 show that apoptotic cells are primarily localized to bronchial epithelial and subepithelial layers, and not to the cells with lymphocyte morphology [38]. Additionally, apoptotic HK483-infected cells are primarily found in the germinal centers of the spleen [38], where CD8 T cells are not typically present in significant numbers. Nevertheless, studies are warranted to assess whether HK483 but not HK486 induces apoptosis of T cells by direct infection. Tumpey et al have reported that the total number of CD8 T cells in the lungs and mediastinal lymph nodes of mice infected with 100 mouse infectious dose 50 (MID_50_) of HK483 was lower than those in HK486-infected mice at day 6 PI [38]. However, in our experiments, contraction in the number of CD8 T cells in the respiratory airways (**Figure 2**) or lungs (data not shown) did not occur until after 8 days after HK483 infection (dose of 18 PFU/mouse or 10 MLD_50_); the number of CD8 T cells in the BAL of HK483-infected mice was lower at day 9 PI, when compared to those in HK486-infected mice. The discrepancy in the kinetics of the CD8 T cell response between the two studies might be related to differences in experimental procedures including preparation of the virus stock, dose of virus used (100 MID_50_ versus 10 MLD_50_), infection procedures, and methods used for isolating mononuclear cells from the tissues.

Despite substantial expansion, virus-specific CD8 T cells were ineffective in controlling HK483 infection, and all mice succumbed within 10 days after infection. The inability of CD8 T cells to effectively control HK483 infection is not associated with functional impairment because virus-specific CD8 T cells in the lung airways contained high levels of granzyme (**Figure 2**) and also produced cytokines, such as IFN-γ, upon antigenic stimulation (**Figure S3**). Additionally, the impaired control of highly pathogenic H5N1 infection is not linked to premature apoptosis of CD8 T cells because protection of CD8 T cells against BIM-dependent apoptosis did not lead to effective viral control or enhanced mouse survival (**Figure 5**). Why is the CD8 T cell response unable to effectively control a lethal H5N1 infection? Recent work suggests that the effectiveness of a CD8 T cell response to successfully control viral replication depends upon the number and concentration of effector CD8 T cells in relationship to the number of virus-infected cells [39], [40]. Therefore, the inability of effector CD8 T cells to control the rapidly replicating HK483 virus might be explained by the large number of virus-infected cells, which leads to higher ratios of effector CD8 T cells to the number of virus-infected cells. The effector CD8 T cell response is perhaps neither fast nor large enough (even in BIM KO mice) to control viruses such as HK483 that are capable of rapid replication and dissemination. Immunotherapies to inflate the number of virus-specific CD8 T cells might be able to control infections with highly pathogenic H5N1 viruses.

The ratio of effector CD8 T cells to virus-infected cells in the tissues could be altered by increasing the number of effector CD8 T cells and/or by decreasing the number of virus-infected cells. Our studies show that oseltamivir treatment can achieve this objective. Oseltamivir therapy at certain doses not only suppressed H5N1 viral titers in the lungs but also enhanced the number of effector CD8 T cells in the lung airways, which in turn led to improved survival. The mechanism(s) underlying the enhancement in CD8 T cell responses by oseltamivir is purely conjecture at this point. One possibility is that oseltamivir reduces viral load, which in turn leads to inhibition of TRAIL induction and BIM-dependent apoptosis of effector CD8 T cells. A second theory is that the diminished viral load in oseltamivir-treated mice would be expected to reduce the amount of HA and HA-triggered cellular apoptosis [41]. A third theory is that CD8 T cell contraction is triggered by extrapulmonary dissemination of the HK483 virus, which elicits a systemic response, and oseltamivir treatment limits viral replication to the lungs. A fourth possibility is that reduced viral load induced by oseltamivir lowered/delayed antigenic stimulation of T cells by DCs, especially early in the infection, which in turn prevented accelerated activation and contraction of CD8 T cells in HK483-infected mice.

It should also be noted that oseltamivir treatment only affected virus titers early in the infection, and viral load in the lungs at the time of T cell contraction (8–9 days PI) was similar in the untreated group as well as in treated groups, regardless of the dose of oseltamivir. These data suggested that viral titers early in the infection might control the contraction kinetics of the anti-viral CD8 T cell response. It has been reported that the early inflammatory response triggered by an infecting organism programs the contraction of CD8 T cell responses [42]. Therefore, the hyperinflammatory response induced by high viral titers early in the H5N1 infection [43] could also be involved in accelerating the kinetics of CD8 T cell activation and contraction in the lungs. Consequently, lower HK483 viral titers induced by oseltamivir treatment would be expected to blunt the inflammatory response thereby delaying the onset of CD8 T cell contraction.

Interestingly, treatment of mice with 10 mg or 20 mg of oseltamivir reduced viral load in the lungs and modulated CD8 T cell responses to a largely similar extent. However, only treatment with 20 mg of oselatmivir led to substantial improvement in survival of HK483-infected mice. In addition to the well-characterized anti-viral effects, the increased survival of mice that received 20 mg of oseltamivir might be explained by at least two non-mutually exclusive mechanisms. First, only treatment with 20 mg or more of oseltamivir can restrict viral replication to the lungs and prevent viral dissemination into tissues like the brain, thereby averting a fatal infection. Second, oseltimivir at this dose might effectively attenuate the host inflammatory response and limit tissue damage by inhibiting pro-inflammatory responses of macrophages [44].

Although cytolytic influenza virus replication alone can cause significant cell death, CD8 T cells are implicated in accentuating tissue damage by immunopathologic mechanisms. We first showed that CD8 T cell deficiency had minimal effects on the survival of mice infected with the highly pathogenic HK483 virus. It is conceivable that in infections with highly lethal viruses, such as HK483, the extremely high rate of viral replication potentially outpaces the innate and adaptive immune responses, and overwhelming tissue damage caused by cytolysis of infected cells is sufficient to cause a lethal infection. A 100% mortality in +/+ mice and the delayed death in CD8 KO mice imply that viral replication is not controlled in +/+ mice, despite the development of a CD8 T cell response.

In summary, in this study, we have defined mechanistic links among the rate of viral replication, viral pathogenicity, the CD8 T cell response, and the clinical outcome of a lethal H5N1 infection in mice. These studies show that the extreme pathogenicity of H5N1 viruses is directly linked to the ability of virus to replicate rapidly and attain high steady-state viral titers in the lungs early in the infection and not due to the lack of a CD8 T cell response. Perhaps, the rapidly replicating virus simply overwhelms and outpaces the most potent CD8 T cell response. Therefore, restraining H5N1 virus replication to levels under a certain threshold early in the infection not only limits direct virus-induced cytopathicity but also allows the development of a CD8 T cell response that can now effectively clear the non-overwhelming infection. These findings have furthered our understanding of the pathogenesis of H5N1 infections and are expected to have significant implications on the development of effective therapies to treat H5N1 infection in humans.

## Materials and Methods

### Mice

6-week-old BALB/c, C57BL/6, BIM KO [45], Fas KO (lpr/lpr) [46], CD8 KO [47], and Clone-4 mice [48] were purchased from Jackson Laboratory (Bar Harbor, ME). The Type I IFNR−/− mice were provided by Dr. Murali-Krishna (University of Washington, Seattle, WA) [49]. All mice were used at 6–8 weeks of age according to the protocol approved by the University of Wisconsin School of Veterinary Medicine Institutional Animal Care and Use Committee (IACUC). The animal committee mandates that institutions and individuals using animals for research, teaching, and/or testing must acknowledge and accept both legal and ethical responsibility for the animals under their care, as specified in the Animal Welfare Act (AWA) and associated Animal Welfare Regulations (AWRs) and Public Health Service (PHS) Policy. Animal experimentation was done as per the PHS Policy on Humane Care and Use of Laboratory Animals as described in the Guide for the Care and Use of Laboratory Animals.

### Virus

HK483 and HK486 viruses that were isolated from patients during the Hong Kong outbreak of 1997 were derived by reverse genetics and titered as described before [13]. Mice were infected I/N with different doses of HK483 or HK486 virus in a volume of 50 µl. Viral titers in tissues were quantified by a plaque assay using MDCK cells. All experiments with these H5N1 viruses were performed in a biosafety level 3 containment laboratory approved for such use by the CDC and United States Department of Agriculture.

### Adoptive transfer of CL-4 TCR transgenic CD8 T cells

Thy1.1/CL-4 CD8 T cells were labeled with CFSE and adoptively transferred into congenic Thy1.2/BALB/c mice by tail vein injection as described before [5]. Twenty-four hours after cell transfer, mice were infected I/N with the HK483 or the HK486 virus.

### Flow cytometry

K^d^/NP147 pentamers were purchased from Proimmune Inc. (FL USA). The D^b^/PA224 tetramers were kindly provided by the NIH Tetramer Facility (Emory University, Atlanta, GA). All antibodies were purchased from BD-Pharmingen unless stated otherwise. Mononuclear cells isolated from BAL or lymph nodes were stained with anti-CD8, anti-LFA-1, anti-CD62L, anti-CD43, and MHC tetramers/pentamers for 1 hr at 4C. For intracellular staining, cells were stained for cell surface molecules as above, and subsequently permeabilized and stained with anti-granzyme (Invitrogen) or anti-caspase 3 antibodies using the Cytofix/Cytoperm kit (BD-Pharmingen). Following staining, cells were fixed with 2% paraformaldehyde and analyzed using a FACSCalibur flow cytometer (Becton Dickinson). Flow cytometry data were analyzed using Flowjo software.

### Histopathology

Mice were euthanized, and tissues were collected and fixed in 10% phosphate-buffered formalin. They were then dehydrated, embedded in paraffin, and cut into 5-µm-thick sections that were stained with standard hematoxylin-and-eosin.

### Oseltamivir treatment

Oseltamivir phosphate (Tamiflu, Roche Laboratories Inc., Basel, Switzerland) dissolved in 50% Ora-Plus Suspending agent (Paddock Laboratories, Inc., Minneapolis, MN, USA) in water and administered to mice once daily by oral gavage in a volume of 200 µL at −1 to 3 days relative to infection with HK483 virus.

## Supporting Information

Figure S1 Induction of IFN-β and IFN-stimulated genes in lungs of HK483-infected mice. BALB/c mice were infected I/N with HK483 or HK486 virus. At day 2 PI, total RNA extracted from lungs were subjected to microarray analyses to determine gene expression profiles using Agilent oligo-nucleotide arrays. Data was analyzed using Rosetta's resolver and SpotFire decision site for functional genomics. Data represent fold increase in gene expression, as compared to uninfected controls.
(0.23 MB TIF)

Figure S2 Viral titers in C57BL/6, FAS KO, and BIM KO mice. Groups of mice were infected with 18 PFU of HK483 virus, and viral titers in the lungs were determined at the indicated days after infection. The data for days 1 and 2 PI are from 2–3 mice/group/time point. Viral titers at days 8 and 9 PI are from 3–12 mice/group.
(0.12 MB TIF)

Figure S3 Interferon gamma production by virus-specific CD8 T cells in the BAL of mice infected with HK483 virus. Groups of BALB/c mice were infected with the indicated doses of HK483 or HK486 virus. Pooled cells from BAL were stimulated for 5 hours with the NP147 peptide, and IFNγ production by CD8 T cells was assessed by intracellular cytokine staining. The FACS plots are gated on total CD8 T cells, and the numbers are the percentages of IFNγ-producing cells of CD8 T cells.
(0.68 MB TIF)
