# Supplementary figures and images for: Viral Replication Rate Regulates Clinical Outcome and CD8 T Cell Responses during Highly Pathogenic H5N1 Influenza Virus Infection in Mice
Source: PLoS Pathog. 2010 Oct 7;6(10):e1001139. doi: 10.1371/journal.ppat.1001139 (PMC2951384; doi:10.1371/journal.ppat.1001139)

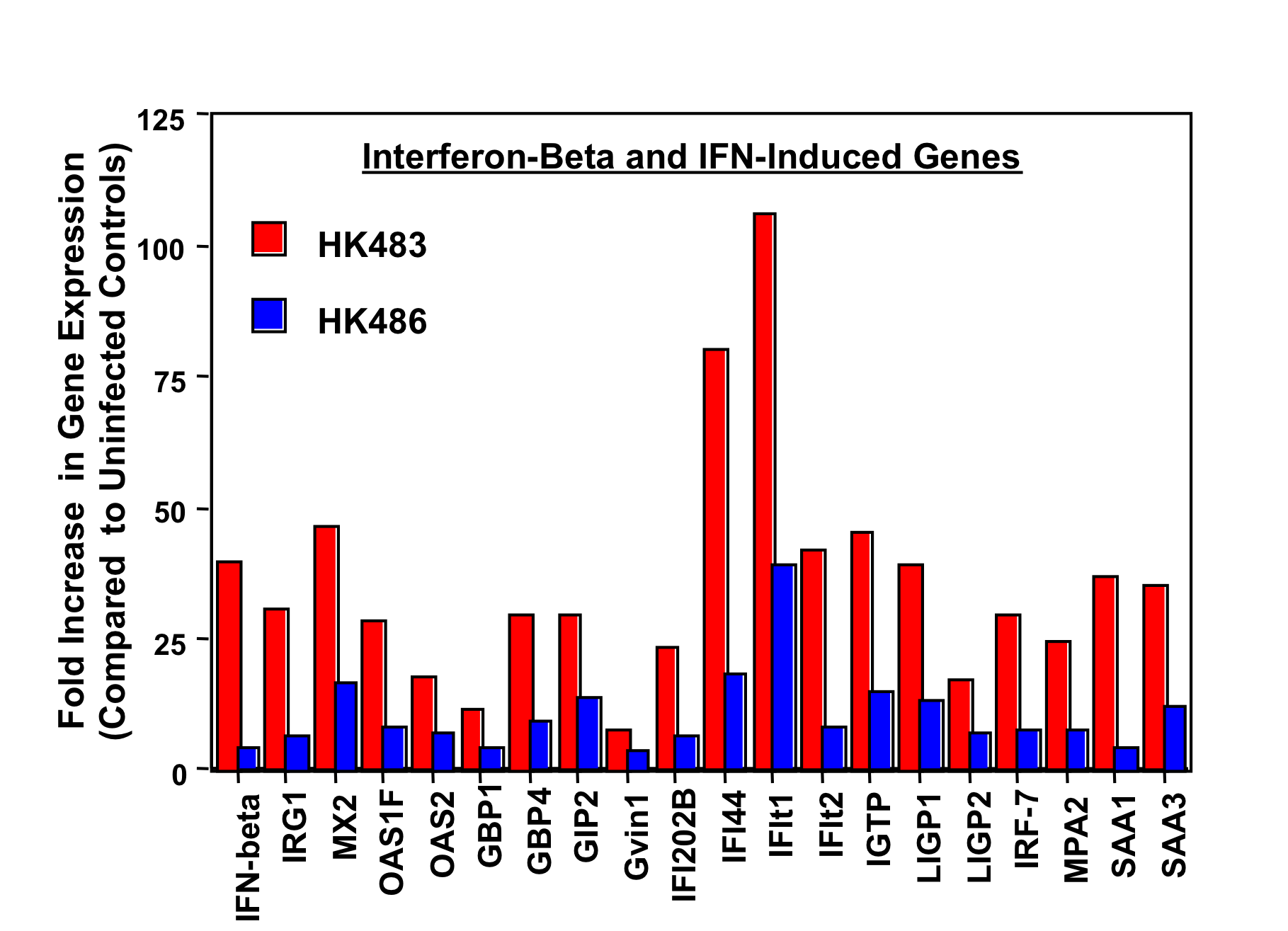

Supplement: Figure S1 — Induction of IFN-β and IFN-stimulated genes in lungs of HK483-infected mice. BALB/c mice were infected I/N with HK483 or HK486 virus. At day 2 PI, total RNA extracted from lungs were subjected to microarray analyses to determine gene expression profiles using Agilent oligo-nucleotide arrays. Data was analyzed using Rosetta's resolver and SpotFire decision site for functional genomics. Data represent fold increase in gene expression, as compared to uninfected controls. (0.23 MB TIF) [file ppat.1001139.s001.tif]

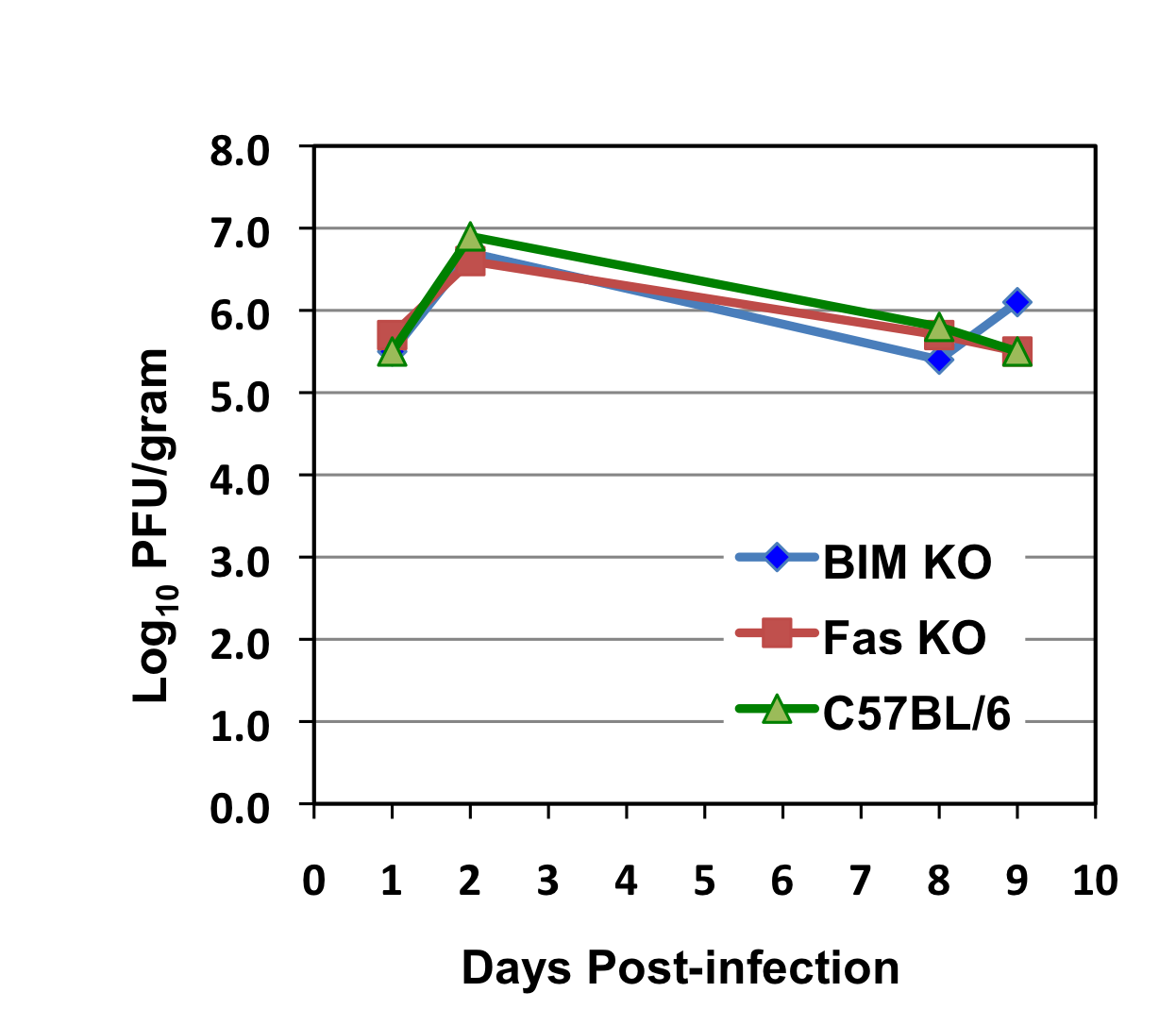

Supplement: Figure S2 — Viral titers in C57BL/6, FAS KO, and BIM KO mice. Groups of mice were infected with 18 PFU of HK483 virus, and viral titers in the lungs were determined at the indicated days after infection. The data for days 1 and 2 PI are from 2–3 mice/group/time point. Viral titers at days 8 and 9 PI are from 3–12 mice/group. (0.12 MB TIF) [file ppat.1001139.s002.tif]

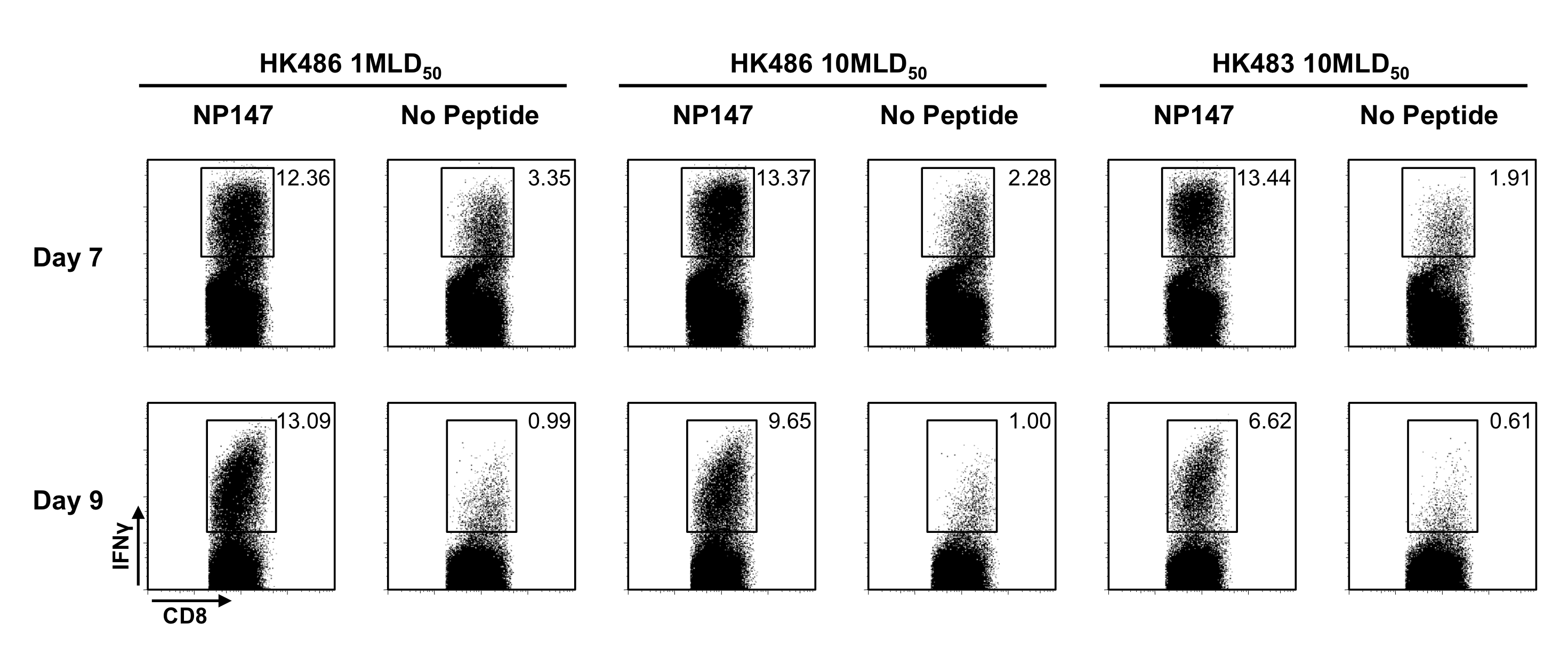

Supplement: Figure S3 — Interferon gamma production by virus-specific CD8 T cells in the BAL of mice infected with HK483 virus. Groups of BALB/c mice were infected with the indicated doses of HK483 or HK486 virus. Pooled cells from BAL were stimulated for 5 hours with the NP147 peptide, and IFNγ production by CD8 T cells was assessed by intracellular cytokine staining. The FACS plots are gated on total CD8 T cells, and the numbers are the percentages of IFNγ-producing cells of CD8 T cells. (0.68 MB TIF) [file ppat.1001139.s003.tif]
